# Damage Detection at a Reinforced Concrete Specimen with Coda Wave Interferometry

**DOI:** 10.3390/ma14175013

**Published:** 2021-09-02

**Authors:** Stefan Grabke, Felix Clauß, Kai-Uwe Bletzinger, Mark Alexander Ahrens, Peter Mark, Roland Wüchner

**Affiliations:** 1 Chair of Structural Analysis, TU München, Arcisstraße 21, 80333 München, Germany; kub@tum.de (K.-U.B.); wuechner@tum.de (R.W.); 2 Chair of Concrete Structures, Ruhr-Universität Bochum, Universitaätstraße 150, 44801 Bochum, Germany; Felix.Clauss@ruhr-uni-bochum.de (F.C.); alexander.ahrens@rub.de (M.A.A.); peter.mark@rub.de (P.M.)

**Keywords:** diffuse ultrasound, coda wave interferometry, structural health monitoring, cracks in concrete, damage detection

## Abstract

Reinforced concrete is a widely used construction material in the building industry. With the increasing age of structures and higher loads there is an immense demand for structural health monitoring of built infrastructure. Coda wave interferometry is a possible candidate for damage detection in concrete whose applicability is demonstrated in this study. The technology is based on a correlation evaluation of two ultrasonic signals. In this study, two ways of processing the correlation data for damage detection are compared. The coda wave measurement data are obtained from a four-point bending test at a reinforced concrete specimen that is also instrumented with fibre optic strain measurements. The used ultrasonic signals have a central frequency of 60 kHz which is a significant difference to previous studies. The experiment shows that the coda wave interferometry has a high sensitivity for developing cracks and by solving an inverse problem even multiple cracks can be distinguished. A further specialty of this study is the use of finite elements for solving a diffusion problem which is needed to state the previously mentioned inverse problem for damage localization.

## 1. Introduction

Concrete is a material commonly used in the construction industry. Especially the combination of concrete and steel reinforcement results in many advantages. During the lifespan of a concrete structure, the appearance of cracks in the structure is very typical. Those cracks are either intentional or due to environmental influences, increased loads, aging or local failure. Regular inspections and assessments are therefore very important to ensure the functionality of concrete structures. In addition to regular visual inspections, permanent structural health monitoring techniques are increasingly used. Established techniques use, for example, strain gauges on concrete and steel.

Coda wave interferometry (CWI) is a rather novel monitoring and damage detection technique applicable to concrete. It is based on elastic waves and originates from geophysics, more precisely, seismology. Larose and Hall [1] were one of the first to apply the technique to concrete and Planes and Larose [2] give a good review on CWI in concrete. The application of CWI to concrete is possible due to the high heterogeneity of the material that creates scattering. This increases the area to which a signal is sensitive and additionally increases the sensitivity to very small changes. The CWI is based on the principle that signals with their diffuse tail created by the scattering can be reproduced. When evaluating a signal, it is compared to a reference signal. As soon as small perturbations appear in the medium, the signal undergoes small changes. An evaluation of these changes in the signal subsequently allows a localization of the cause that are often cracks in the concrete.

For concrete structures, the used signals have a central frequency in the ultrasonic spectrum. Planès and Larose [2] differ the single scattering regime with frequencies between 20 and 150 kHz and the multiple scattering regime with frequencies between 150 kHz and 1 MHz. In general, an increase of the signal scattering improves the CWI performance. This is accompanied by the use of higher frequencies, which, however, reduces the maximum possible distance between the source and receiver. Larose et al. [3] and Zhang et al. [4,5] have successfully applied CWI for damage detection in real concrete structures with frequencies in the multiple scattering regime. A more recent experiment of Zhan et al. [6] and Jiang et al. [7] has conducted a similar, inverse problem based, imaging and successfully detected multiple cracks whose position, however, strongly correlates with the ultrasound sensors that were attached to the surface after cracking. On a structural scale, several groups [8,9,10,11,12,13] have conducted field experiments with CWI and demonstrated the immense potential of the technology based on an signal evaluation but without a damage localization as it is done in the present study. For the monitoring at a very large, structural scale, greater measuring distances are required. Thus, this study tests the use of 60 kHz that in concrete rather belongs to the single scattering regime but based on Fröjd and Ulriksen [14] should be applicable for CWI in concrete. For this purpose, CWI is applied to a four-point bending test on a reinforced concrete specimen.

In Section 2, the general principle of CWI is described and two methods for the localization of damage are introduced. A special and novel feature of the first damage localization approach is a substitute model which is based on a finite element simulation. The second approach to damage localization is a very simple, novel technique based on the arithmetic mean, that in contrast to the first approach does not require the solution of an inverse problem an thus is very fast. Section 3 gives an overview of the experiment with the used test set-up and expected behaviour of the material. Next to ultrasound measurements, strain in a reinforcement bar is measured with fiber optic sensors (FOS). In Section 4, the CWI data are analysed and a damage localization is performed at different damage states. For the localization, the two used ultrasound based imaging techniques are compared to the FOS data. In the end the damage localization results with CWI at the four-point bending test are discussed.

## 2. Ultrasound Methods

Coda wave interferometry uses diffuse ultrasound to measure relative changes of a signal compared to a reference state. Those changes are typically created by a change of the concrete’s temperature [15], moisture [16] and stresses [1,17] that mostly affect the wave speed of the signal but also changes in the propagation medium due to cracks modify the signal [18]. If none of these changes occur in the medium, signals and their diffuse tails can be reproduced. This study puts a focus on damage detection that requires a CWI specific signal processing described in Section 2.1 which then allows to localize damage.

### 2.1. Basics

The central measurement parameter in CWI is a cross-correlation coefficient (CC) that quantifies the similarity of two compared signals. It is computed for a time frame of length *T* in the signal φ at time *t* as following [19,20]:

(1) $$CC\left(t\right)=\frac{\int_{t-T/2}^{t+T/2}\varphi_{ref}\left(t\right)\varphi \left(t\right)\;dt}{\sqrt{\int_{t-T/2}^{t+T/2}\varphi_{ref}^{2}\left(t\right)\;dt\int_{t-T/2}^{t+T/2}\varphi^{2}\left(t\right)\;dt}}$$

Very often, the *CC* is translated into a decorrelation coefficient (*DC*) that uses a magnitude from zero to one to describe how large the changes are in a signal. The relation of *CC* and *DC* is as follows:

(2) DC=1−CC

The decorrelation of two signals is used for imaging the cause of the signals’ changes. Depending on the used method, the signal is either evaluated in one long time frame or multiple successive shorter time frames. Multiple evaluation windows can evaluate a DC development that tends to increase in later parts of the signal because more random wave paths cross the new scatterer and create different interferences with other wave fronts that all add up to an increased decorrelation. This described increasing development is very characteristic for the relative position of a cause to the source-receiver pair and is modeled by the sensitivity kernel introduced in Section 2.3. With this substitute model, an inverse problem can then be formulated whose solution localizes the cause of the changes. When evaluating only one time frame per measurement, one DC can be assigned to each measurement pair. With the use of influence areas for each pair (Section 2.5) the decorrelations can then be mapped on the geometry.

With an ongoing monitoring, there are multiple measurements performed with each pair. For choosing a reference, there are typically two possibilities. One is a fixed reference measurement, e.g., the first one. The other one is a stepwise update of the reference signal such that the used reference is always the previous measurement. The CWI is based on small changes in the signal and a general reproducibility of the signals. This is usually fulfilled with the stepwise updated reference approach but not necessarily with a fixed reference. Thus, the stepwise approach is chosen for this study.

The measured decorrelations are typically created by two slightly different phenomenons. One is a waveform distortion and the other one is a phase-shift of the signal. The phase-shift is created by a change of the signals’ wave speed that is inducted, e.g., by the acoustoelastic effect [17] that links wave velocity and stresses in the medium. Waveform distortions are typically caused by new scatterers such as cracks. With a focus on damage detection, the impact of phase-shifts on the decorrelation should be minimized by stretching the signal with a stretching factor ε. This cancels out the phase-shifts and is done with the technique introduced by Sens-Schönfelder and Wegler [21]. After stretching, the remaining decorrelation of two compared signals is assumed to be mainly caused by new scatterers such as cracks.

**Figure 1:**
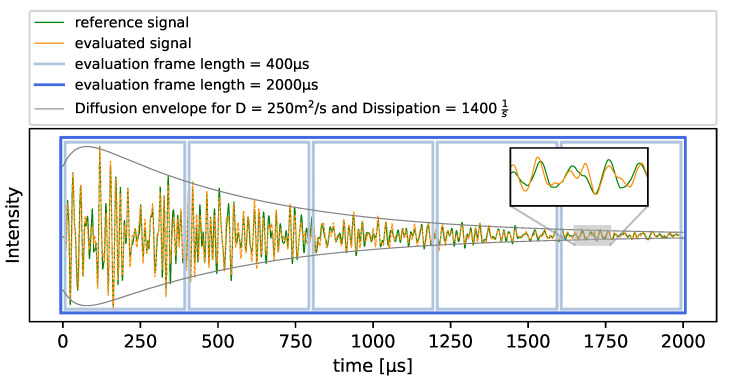
Example signal (60 kHz) with the used evaluation windows and envelope fitting with the solved diffusion equation.

In this study, signals with a length of 2000 μs after the signals’ time-of-flight (tof) are evaluated. For computing one overall DC of a measurement, the time frame is of length T=2000μs. When evaluating a DC development within the signal, five successive time frames with length T=400μs are used. This is shown in Figure 1.

### 2.2. Diffusion Approximation

When thinking of a simulation that resembles the performed experiment, the model would need to contain a heterogeneous material with a very fine refinement in order to represent the used concrete. In addition, the time steps used would have to be very short to achieve numerical stability for an acoustic wave simulation. This causes great computational costs that limit the maximum size of the modeled geometry. Thus, a central simplification is used in the description of how the wave propagates through a heterogeneous medium. As Ryzhik et al. [22] have shown, the spread of a waves’ energy in a random media as concrete can be approximated with a diffusive spread in a homogeneous medium. This significantly improves computational costs. The main parameter of this approximation that includes the overall scattering behavior of concrete is the diffusivity *D*. It can be determined with an envelope fitting of the signal as shown in Figure 1 where one can see the relation of the complex, diffuse signal and the simple diffusion envelope. Doing so, the mean diffusivity in this study was determined at about 250 m^2^/s.

This study uses a novel finite element (FE) based formulation that solves the given problem. The use of FE is a significant difference to previous applications that are usually based on analytical solutions and opens the door to many improvements of the technology. FE and the accompanying use of unstructured meshes allow the problem to be solved for arbitrary, complex-shaped geometries, and the generic FE approach even allows the solution to be further improved by using a different partial differential equation that better approximates the given wave phenomenon. The present study uses the open-source project KRATOS Multiphysics (www.cimne.com/kratos) for solving the FE problem.

### 2.3. Sensitivity Kernel

For simulating the actual correlation measurements of two compared signals, a substitute model introduced by Pacheco and Snieder [23] is used that computes sensitivities of a measurement to a local change. This so called sensitivity kernel describes the possibility that a wave has passed a location *x* during a travel time *t* from the source *S* to the receiver *R* and thus describes where a wave got its information from. It is calculated as follows:

(3) $$K\left(S,R,x,t\right)=\frac{\int_{0}^{t}I\left(S,x,u\right)I\left(x,R,t-u\right)\;du}{I(S,R,t)}$$

In Equation (3), I(pos1,pos2,t) stands for the wave intensity which here is equated to the possibility of a wave travelling from pos1 to pos2 in time *t*. Instead of an actual wave intensity, the approximation of Section 2.2 is used and the FE solution of the simulation with KRATOS Multiphysics is inserted for *I*. At each position *x* the sensitivity kernel gives a development over time that resembles the DC development over the signals’ length in case a scatter is added at the corresponding position *x*.

### 2.4. Imaging with an Inverse Problem

Being able to simulate the DC development for any location *x* allows to formulate a problem that, when solved, localizes the cause for the decorrelation. Planès et al. [24] describes this problem as

(4) Gm=d

where *G* is a matrix that contains the sensitivity kernel for one specific measurement pair at a specific time in each row. The vector *d* contains the measured decorrelation in the signal with pair and time matching the sensitivity kernel in the corresponding row. The vector *m* contains the damage at each node of the mesh and is the unknown in this equation. The size of *G* is *n × m* with *n* referring to the amount of nodes in the mesh and *m* referring to the total amount of measurements. Typically the amount of nodes in the mesh is larger than the amount of measurements and thus, the problem is underdetermined. The equation system of Equation (4) is inconsistent and reformulated to a least-squares optimization problem:

(5) $${\min∥d-Gm∥}_{2}$$

For solving this large-scale ill-posed problem, a trust region reflective algorithm by Branch et al. [25] called STIR is used in this study. It is referred to as a subspace, interior and conjugate gradient method for bound-constrained minimization problems. Especially the boundary on the variables is very useful since damage effects in the coda signal can only add up (no negative values allowed) and the maximum effect of one node in the mesh should also be limited to $m_{max}$.

### 2.5. Imaging with Influence Areas

**Figure 2:**
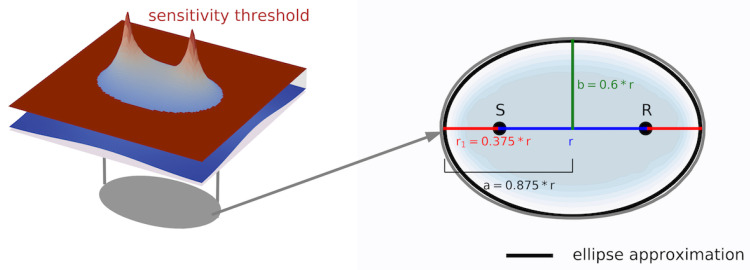
Graphical explanation of the derivation of the influence areas with the help of a sensitivity kernel.

Next to a simulation that should resemble the DC measurements, a second, simpler approach is used in this study. For this novel approach, an influence area is assigned to each measurement pair. In order to choose such an area, a signals sensitivities computed with the sensitivity kernel are used. The used sensitivity kernel has a source-receiver distance of 30 cm which resembles the typical distance used in the experiment that is introduced in Section 3. However, a different source-receiver distance of the kernel produces a similar looking sensitivity kernel and thus the approach can be used for other source-receiver distances as well. By limiting the sensitivities to a minimum threshold, a nearly elliptical area is obtained. The ellipse is transferred to a generic description that depends on the source-receiver distance *r* which is visualized in Figure 2. In this study, an ellipse with the semi-major axis a=0.875∗r, the semi-minor axis b=0.6∗r and an eccentricity e=r/2 is used. The obtained ellipse parameters are depending in the chosen sensitivity threshold which is a free parameter. Thus, the influence areas can be varied in case the obtained imaging has too strong contrasts or if the smoothing is to be reduced. In order to obtain a smooth overlap with other regions, the influence vanishes to the ellipse border, which is indicated by blue on the right of Figure 2.

With the influence areas, the transfer of measured decorrelation to a spatial representation on the geometry is done with the weighted arithmetic mean. The DC at a position *x* is obtained as follows:

(6) $$DC\left(x\right)=\frac{\sum_{p}DC\left(p\right)*w\left(x,p\right)}{\sum_{p}w\left(x,p\right)}$$

where w(x,p) is the influence of a pair *p* at a position *x* and DC(p) the overall decorrelation per pair evaluated with a frame length T=2000μs.

## 3. Experiment

**Figure 3:**
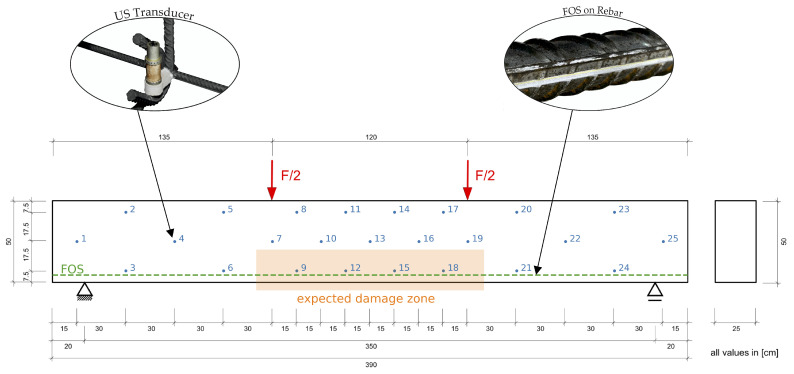
Dimensions of the test specimen with FOS in green and US transducers in blue. Results of the US transducer 16 proved to be erroneous and were not taken into account for the evaluation.

In order to prove the stated imaging on both the inverse problem and the influence areas, a structural test was carried out at the Ruhr University, Bochum, Germany. The reinforced concrete specimen is a beam with a depth of 500 mm, width of 250 mm and length of 3900 mm. Resulting from the loading in a 4-point bending test, flexural reinforcement (3Ø20 mm) as well as staggered stirrup reinforcement (Ø12 mm / 300 mm / 2) are placed. With a field length of 3500 mm (cf. Figure 3) and a spacing of the two concentrated loads of 1200 mm, this system represents a challenging way to demonstrate imaging. Substantiated by regularly occurring cracks between the concentrated loads. These cracks, which appear in multiplicity, unlike a single very local crack, render it difficult for the algorithm to output detailed predictions. Thus, the system provides a good means to prove the algorithm’s performance.

To detect cracks, an ultrasonic signal is intentionally to be guided into the concrete. For this purpose, ultrasonic transducers (SO807 transducers from Acoustic Control Systems, Ltd., Saarbrücken, Germany) embedded in the concrete are arranged in a net-like manner throughout the specimen (cf. Figure 3). The aim was to cover the whole specimen with the sensor-net to be able to see differences of cracked and intact regions. With expected cracking in the middle third the density of the sensor-net was increased in this part to be able to closer investigate the effects of cracks on different measurement pairs. The transducers, consisting of a piezoceramic cylinder, exhibit a central frequency of approx. 60 kHz. The radiation takes place almost uniformly, perpendicular to the longitudinal axis of the transducer. Cement fasteners are used to attach the transducers to the reinforcement. Due to the similarity of the cement fasteners to the surrounding concrete, the fastener’s influence on the load-bearing behavior can be prevented. During the evaluation process it became evident that transducer 16 is erroneous and thus related measurements were not taken into account.

In addition to ultrasonic measurement technology, FOS were used in particular. The FOS technology provides the possibility to measure strains and temperatures [26,27] with high resolution (point distance about 0.65 mm). For this experiment, the Luna ODiSI 6008 fiber optic instrument (Roanoke, TX, USA) was used. A FOS was glued to a rebar (Ø 20 mm). The application of the fiber onto the rebar was realized by the adhesive Polytec PT AC2411 (Karlsbad, Germany). This adhesive proved to be suitable in detailed investigations [28] into the adhesive and fiber to be used.

**Figure 4:**
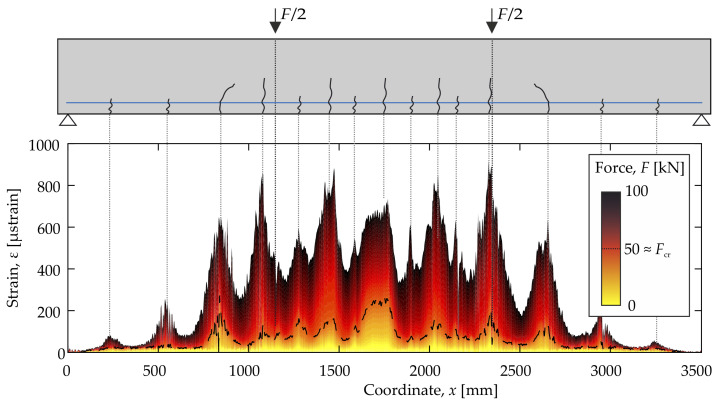
Strain results from 0 to 100 kN of the FOS. Due to the continuous measurement, the colors are referring to the load of the corresponding strain measurement.

From the very continuity of the fiber optic strain measurement (both temporal and spatial), it becomes evident that a lot of strain data are acquired. The measured strains along the rebar for a load from 0 to 100 kN is shown in Figure 4. The increasing force is reflected in the (color) profile of the strains.

Due to the low tensile strength of concrete, it cracks even when subjected to a low tensile stress. The tensile strength $f_{ctm}$ given in Table 1 corresponds to the mean value from three individual samples. Variations around this mean value are a matter of course and, like many other natural processes, can be regarded as a log-normal distribution. Consequently, it is probable that cracking (an excess of the scattering tensile strength) begins earlier than (<$f_{ctm}$) the given mean value. If concrete cracks, the force, which was previously carried by the entire intact cross-section, is transferred to the reinforcing steel. The strain or stress of the reinforcing steel thus rises in the crack. As the distance to the crack increases, force is transferred back into the concrete via the bonding effect of the reinforcement and the concrete. When sufficient force is again transferred into the concrete and the tensile strength is again exceeded, the next crack forms. With complete formation of the crack pattern (>$1.3F_{cr}$), the peaks and valleys in the strain profile shown in Figure 4 appear. This defining characteristic of the load-bearing behavior of reinforced concrete can be used to capture the development of cracks and serves as a reference for the used ultrasonic measurement technology.

## 4. Results

The evaluation of the CWI data is performed in two different ways. Section 4.1 analyses the overall DC development of selected signals during the whole load increase process and Section 4.2 performs a damage localization at three different load states.

### 4.1. Decorrelation Investigations on Selected Measurement Pairs

**Figure 5:**
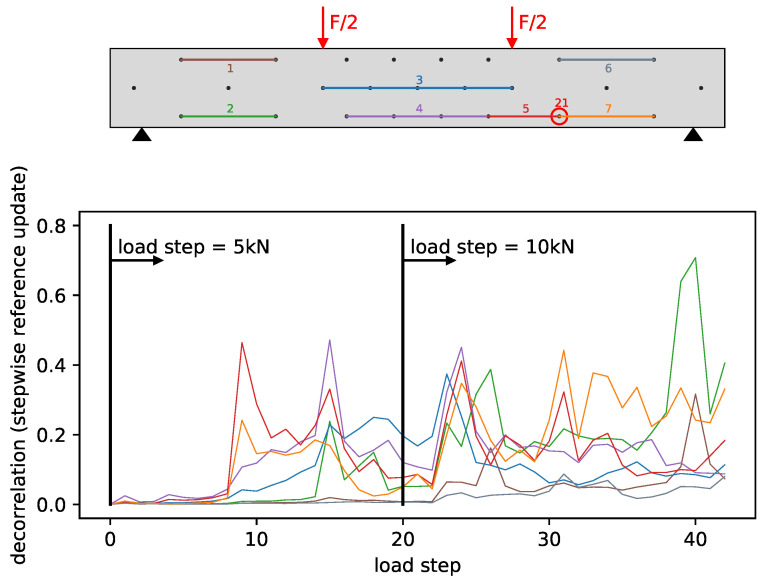
Decorrelation development on selected measurements.

Figure 5 shows the DC development of seven selected measurement pairs. The aim of this selection is to cover areas in the structure with different stresses. The following paragraphs group comparable pairs with the numbering relating to Figure 5.

#### 4.1.1. Pair 1 and 6

Pair 1 and 6 are in a zone with comparable small stresses that is also the furthest away from developing cracks. The DC development with values around 0 up to load step 22 confirms this. Further load increases generate a slight increase of the DC that is relatively small compared to other measurement pairs. The reason are probably massive cracks in the middle third and a crack development further to the sides. This shows that measurement pairs have a limited extended sensitivity and the influence of ballistic waves passing through the specimen several times is very small.

#### 4.1.2. Pair 3 and 4

Pair 3 and 4 are in the zone with the highest tension stresses and an area where multiple cracks are expected to develop. The DC development shows a first significant increase in the DC around load step 8 and continues to increase until approximately load step 16. This indicates strong crack formation. The constant increase of the DC can be explained by the fact that the cracks open up more and more and thus, the transmission of the ultrasonic waves becomes increasingly worse. The time of crack formation coincides approximately with the average tensile strength of the concrete and is closer investigated in Section 4.2.2.

#### 4.1.3. Pair 5 and 7

**Figure 6:**
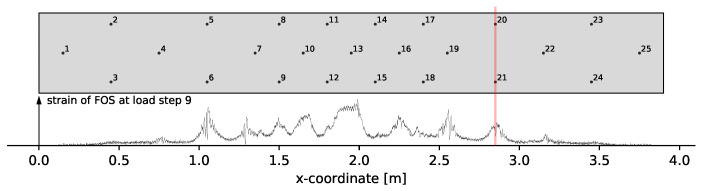
Comparison of FOS data to transducer positions at load step 9.

Pair 5 and 7 are closer investigated due to an observation that led to problems in the damage localization. Figure 6 shows the FOS data at load step 9 next to the transducer positions.

The FOS indicates cracks close to transducers 6, 9 and 21 at load step 9. For transducer 21 at x = 2.85 the formed crack seems to have a large impact on the general reproducibility of the signals related to that transducer. It is possible that a crack into the mounting position of transducer 21 massively impacts the transmission of ultrasound into the concrete. This generates unusually large decorrelation. The fact that the decorrelation is unusually large also becomes apparent when comparing pair 2 and 7. Furthermore, the DC of pair 5 and 7 would be expected to be smaller than for pair 4. The unusual large decorrelation becomes a problem for the damage localization since the modeled approximation of measurements is not valid anymore. Affected measurements are therefore filtered from the used measurement set.

### 4.2. CWI Damage Localization

For the damage localization, three states are distinguished. One is before cracks are detected by the FOS (Section 4.2.1), one is during the formation of first cracks (Section 4.2.2) and the last one is after the average tensile strength is reached (Section 4.2.3). For all three states, the FOS data are compared to a damage localization by solving an inverse problem (Section 2.4) and the spatial representation of the correlations with the help of influence areas (Section 2.5).

#### 4.2.1. State 1: Uncracked

**Figure 7:**
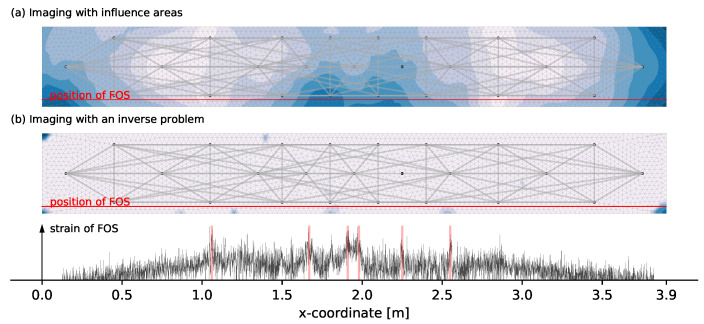
Evaluation at load step 3 with 15 kN load applied with the two different methods of imaging from Section 2.4 and Section 2.5 shown in (**a**,**b**).

In the early load stages, it is expected that the different monitoring systems detect no damage. An evaluation of load step 3 at 15 kN shown in Figure 7 generally confirms this assumption but indicates first tendencies. Those tendencies are the formation of first little peaks in the FOS (marked with red color in Figure 7) and an imaging of the overall decorrelation with the influence areas that among others tends to the tension zone in the middle. Those first tendencies of the coda correlations underline the high sensitivity of the technique to very little changes in the medium such as micro cracks. The high values in the lateral edge areas of Figure 7a are presumably related to the low density of measurement pairs.

**Figure 8:**
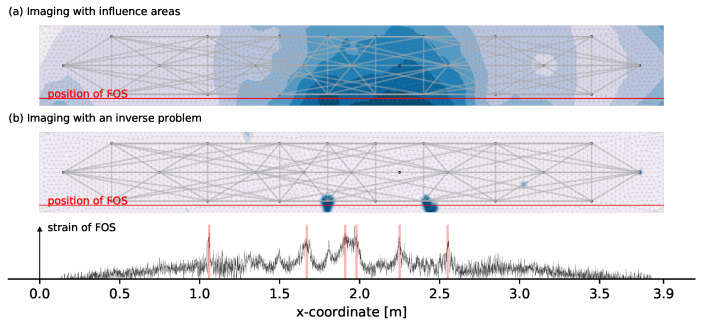
Evaluation at load step 4 with 20 kN load applied with the two different methods of imaging from Section 2.4 and Section 2.5 shown in (**a**,**b**).

An evaluation at the next load step with 20 kN applied confirms the first trends from the previous load step. Especially the first tendencies of the FOS data in Figure 7 that are marked in red have now become clearly visible peaks. For comparison, the six positions marked in red in Figure 7 are also shown in Figure 8. One can see that the high values in the lateral edge regions of the influence areas imaging have nearly vanished presumably because decorrelation in the tension zone is clearly greater. Additionally, one can notice that the tension zone shows the largest decorrelation but also the top middle part indicates large DC values. Besides the expansion of the influence areas, this is presumably related to the larger changes of the stress state compared to the other areas. At this load step, where the first cracks are clearly detected by the FOS, the solution of the inverse problem shows first tendencies as well. However, the localization does not match the FOS data and rather determines the center of several small cracks. This load state with a manageable crack pattern is used to determine the magnitude of the solution and the maximum boundary $m_{max}$ for the inverse problem. A value of $m_{max}=2$ shows a reasonable crack extension and is thus used for an evaluation at all other load steps.

#### 4.2.2. State 2: Crack Formation

**Figure 9:**
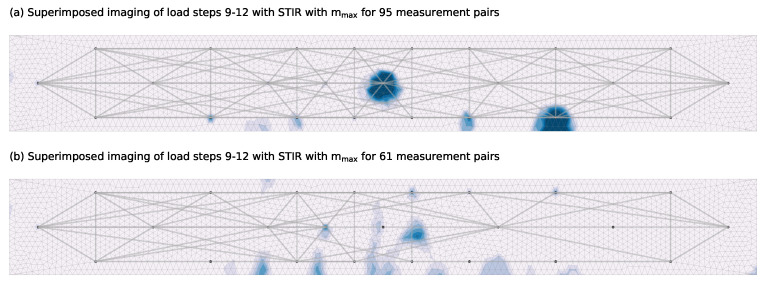
Comparison of the damage localization with an inverse problem for 95 pairs (**a**) and DC-based selected 61 pairs (**b**) using the STIR method with $m_{max}=2.0$.

Load step 4 shows that the first micro cracks already occur at stresses significantly below the average tensile strength of the concrete used $f_{ctm}$, which is reached at load step 10. This shows that a clear determination of the first real damage is difficult. For an evaluation of the crack formation, the load steps 9 to 12, which are around the calculated crack formation, are closer investigated. A comparison of these consecutive load steps 9–12 shows similarities in the detection but also clear differences at certain located crack positions can be noticed. A possible reason could be a non-uniform crack development. In order to reduce such influences, the imaging shown in Figure 9 superimposes the four individual damage images of load steps 9 to 12 which are the load steps where the first cracks are most likely to develop. Adding up the damage is justified insofar as the stepwise reference update as described in Section 2.1 is used and the superposition then represents the change from load step 8 to 12. In Figure 9a one can see that in general, the positions of several transducers are detected as most likely damage locations. By exploiting the symmetry of the experiment, unusually large decorrelation developments for certain measurement pairs as they are also described in Section 4.1.3 can be noticed. These unusually large decorrelation measurements are filtered from the total set. The remaining measurement net of 61 pairs with the corresponding superposed imaging of load steps 9 to 12 is shown in Figure 9b.

**Figure 10:**
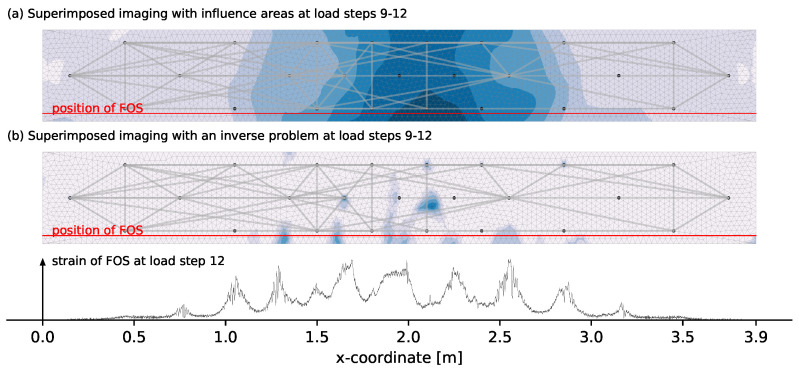
Evaluation of the change between load step 8 and 12 with the two different methods of imaging from Section 2.4 and Section 2.5 shown in (**a**,**b**).

The image from Figure 9b looks insofar good that transducer positions are generally no longer detected as most likely damage location. Figure 10 compares the results to the influence areas imaging and FOS data. The influence areas imaging indicates significantly larger decorrelation for the middle third as it is expected. The extension of this middle area is also in relative good accordance to the extension of the crack pattern determined with the FOS. Large changes over the height can only be detected to a limited extent, which is probably related to the relatively large sensitivities of the upper measurement pairs for the bottom area and the use of vertical and diagonal measurement pairs.

The crack pattern obtained with the inverse problem shows a good correlation with the more reliable FOS data. Remarkable is the distinction of multiple cracks which is strongly influenced by three factors. One is the superposed imaging. Each of the four individual damage images has a tendency to certain cracks or areas. Superimposing multiple states smoothes out this observation that possibly is related to a non-uniform crack development between each load step. The second factor is the use of an upper boundary on the variables of $m_{max}=2$ for the given set-up. This prevents a localization at the center of multiple cracks. The last factor is the pre-selection of used measurements pairs based on unusually large decorrelation development whose impact can be seen in Figure 9.

#### 4.2.3. State 3: Cracked

**Figure 11:**
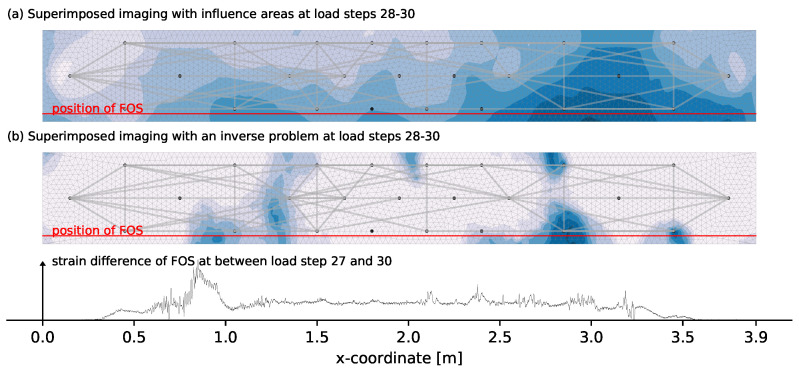
Evaluation of the change between load step 27 and 30 with the two different methods of imaging from Section 2.4 and Section 2.5 shown in (**a**,**b**).

The third evaluation state is chosen relativity late in the loading progress. The aim is to investigate the extent to which a crack pattern still changes and what effect this has on the coda measurement technique. As in Section 4.2.2 several measurement pairs are taken from the set of used pairs because with those measurements mainly installation positions of transducers are localized as damage location. Figure 11 shows the detected changes between load step 27 and 30.

The FOS data and also the inverse problem imaging show that the crack development in the middle third is completed and changes in the specimen now appear in the previously unaffected areas further to the sides. This underlines the fact that existing cracks can only be detected to a limited extent with CWI. Once a crack development is completed, the CWI with a stepwise updated reference will not detect that crack since no more changes appear in the medium. The FOS data shows one clearly visible peak on the left and also the inverse problem localizes that area. Additionally, there are a few other areas detected that can not be seen in the FOS data. This can have two possible causes. First one is that the crack development at the height of the FOS is completed and a crack only develops above the FOS mounting height. The second one is that the filtering of unusually large decorrelated measurement pairs by engineering instinct was not sufficient. A further filtering would, however, affect an equally balanced measurement net. Thus, the conclusion is that in such a highly cracked condition, the application of CWI in concrete is very difficult.

## 5. Discussion

### 5.1. Overall Discussion with an Outlook to General Improvements

The material behavior in the four-point bending test is as expected. The FOS data appear to be very reliable and detect multiple cracks at a very early load state. Its crack detection, however, is limited to one reinforcement bar only and the installation on a large structural scale shows problems since the careful application of the fibre on the reinforcement is labour intensive and thus expensive. Here are advantages of the CWI that shows the potential to detect multiple cracks and covers a larger area with a simpler installation. Despite a longer wavelength compared to previous experiments by Larose et al. [3] and Zhang et al. [4,5], the sensitivity of the CWI is remarkably high. The damage localization by solving an inverse problem for the time of crack formation as shown in Figure 10 is of good accuracy compared to the FOS data and especially the distinguishing of multiple close cracks is remarkable. The comparable experiment of Zhan et al. [6] and Jiang et al. [7] achieved similar results but with the very close positions of cracks and transducers the problem of the present study with a detection at transducer positions (cf. Figure 9) can not be excluded. The used frequency of 60 kHz is a novelty in damage localization with CWI in concrete and opens the door for an application on a large structural scale with longer distances between source and receiver. The present experiment used a rather dense measurement net as the aim was to localize multiple cracks as exact as possible in a controlled environment. When applying CWI on large structures, e.g., bridges, and the aim is to trigger an alarm when cracks in concrete appear but need no precise localization, the transducer network can be chosen less dense and with larger source-receiver distances to cover the whole structure. However, estimating transducer density and localization accuracy is quite difficult and part of ongoing research. Experiments by Wang et al. [10,29] and Fröjd and Ulriksen [30,31] demonstrated the applicanility of CWI with source-receiver distances of at least 1 m. In the present experiment transducers are embedded into the concrete which ensures good transmission of the ultrasonic signal into the concrete and general robustness. As several experiments [4,5,6,32] have shown, the use of external transducers attached to the surface is also possible and allows monitoring of existing structures.

### 5.2. Crack Detection and Related Challenges

The exact definition of one load step at which cracking happens is rather difficult. Thus, several load steps around the load level at which the concretes tensile strength is reached are evaluated and superimposed. With comparably large changes in the structure, the CWI is susceptible to bad measurements in the dataset. Those bad measurements can be caused by cracks into the mounting position of transducers. For the given specimen, this is not unusual because the ultrasound transducers weaken the cross section and thus attract cracks. Related measurements need to be filtered from the used dataset since the DC are unusually large and do not match used assumptions, e.g., a general good reproducibility of the signals. To date, this is done with engineering instinct, but will be parametrized and automated in future studies. A statement about the size of the cracks is not yet possible. The goal of future research will be to find structure-related damage limits for the coda technology. The analysis in the cracked state revealed a weakness of the CWI that existing damage is difficult to detect with a stepwise updated reference.

### 5.3. Comparison of Imaging Approaches

Comparing the two CWI imaging approaches, the solution for an inverse problem can locate damage locations better than what is possible with influence areas. The computational effort however is significantly larger since an ill-posed problem needs to be solved whereas with influence areas only a matrix vector product is computed. Nevertheless, the influence areas have proven to be a useful tool for transferring the measured decorrelation to a spatial representation on the geometry. Especially with a lot of overlapping of measurement pairs, the technique is advantageous to simpler interpolations as, e.g., used by Niederleithinger et al. [8]. The extension of highly “decorrelated” regions was also in accordance to computed stresses and crack regions. For future research, it would be conceivable to use the fast mapping with influence areas as an initial guess for the inverse problem.

The FE formulation for the given problem is a major difference compared to previous studies [4,5,6,7] that use an analytic solution of the diffusion problem or the radiative transfer equation. The generic FE approach is based on unstructured meshes and allows for several improvements such as an application to arbitrary, complex geometries as it is needed when transferring the technology to real structures such as bridges.

## 6. Conclusions

Overall the present study shows the high potential of CWI as a monitoring and damage detection technique for large structures. The used set-up with a central frequency of 60 kHz that belongs to the single scattering regime is a novelty in for inverse problem based damage detection with CWI in concrete. The high sensitivity to small changes in the concrete as well as a good localization and distinguishing of multiple appearing cracks underline the immense potential of CWI in concrete. The addressed problem of unusually large decorrelations that do not fit the model of the inverse problem (sensitivity kernel) in a suitable way and thus hinder a good localization is a challenging problem that was solved in a rather simple way by removing related measurements from the problem to be solved. The finite element based methodology is a significant difference and improvement over established approaches that use analytical solutions. The use of a new solving technique that contains boundary constraints for the inverse problem is another presented novelty that significantly improves the imaging results. The presented imaging using influence areas marks a new, simpler but less accurate way of imaging CWI results that is more independent of a good match between the model and measurement data and thus a more robust way of imaging. In summary, the frequency range used in combination with finite element based localization has proven to be suitable for damage localization in concrete with CWI and the experiment and used methods provide a reliable basis for upscaling the technology to large existing structures.

## Figures and Tables

**Table 1:** Material properties of the concrete.

$f_{\mathrm{cm},\mathrm{cube}}$	$f_{\mathrm{ctm}}$	$E_{\mathrm{cm}}$
$\left[N/{\mathrm{mm}}^{2}\right]$	$\left[N/{\mathrm{mm}}^{2}\right]$	$\left[N/{\mathrm{mm}}^{2}\right]$
38.2	2.8	28,800

## Data Availability

Data sharing not applicable.

## Abbreviations

The following abbreviations are used in this manuscript:

CWI	Coda Wave Interferometry
CC	Cross-Correlation Coefficient
DC	Decorrelation coefficient
FOS	Fibre Optic Sensor
FE	Finite Elements

## References

[1] Larose E., Hall S. (2009). Monitoring stress related velocity variation in concrete with a 2.10^−5^ relative resolution using diffuse ultrasound. J. Acoust. Soc. Am..

[2] Planès T., Larose E. (2013). A review of ultrasonic Coda Wave Interferometry in concrete. Cem. Concr. Res..

[3] Larose E., Obermann A., Digulescu A., Planès T., Chaix J.F., Mazerolle F., Moreau G. (2015). Locating and characterizing a crack in concrete with diffuse ultrasound: A four-point bending test. J. Acoust. Soc. Am..

[4] Zhang Y., Planès T., Larose E., Obermann A., Rospars C., Moreau G. (2016). Diffuse ultrasound monitoring of stress and damage development on a 15-ton concrete beam. J. Acoust. Soc. Am..

[5] Zhang Y., Larose E., Moreau L., d’Ozouville G. (2017). Three-dimensional in situ imaging of cracks in concrete using diffuse ultrasound. Struct. Health Monit..

[6] Zhan H., Jiang H., Jiang R. (2019). Three-dimensional images generated from diffuse ultrasound wave: Detections of multiple cracks in concrete structures. Struct. Health Monit..

[7] Jiang H., Zhan H., Ma Z., Jiang R. (2020). Comparative Study of Three-Dimensional Stress and Crack Imaging in Concrete by Application of Inverse Algorithms to Coda Wave Measurements. Sensors.

[8] Niederleithinger E., Wang X., Herbrand M., Müller M. (2018). Processing Ultrasonic Data by Coda Wave Interferometry to Monitor Load Tests of Concrete Beams. Sensors.

[9] Wang X., Niederleithinger E. Coda Wave Interferometry Used to Detect Loads and Cracks in a Concrete Structure Under Field Conditions. Proceedings of the 9th European Workshop on Structural Health Monitoring Series.

[10] Wang X., Niederleithinger E., Chakraborty J., Klikowicz P. Monitoring a concrete bridge girder with the coda wave interferometry method. Proceedings of the 5th International Conference on Smart Monitoring, Assessment and Rehabilitation of Civil Structures.

[11] Wang X., Chakraborty J., Bassil A., Niederleithinger E. (2020). Detection of Multiple Cracks in Four-Point Bending Tests Using the Coda Wave Interferometry Method. Sensors.

[12] Zhan H., Jiang H., Zhang J., Jiang R. (2020). Condition Evaluation of an Existing T-Beam Bridge Based on Neutral Axis Variation Monitored with Ultrasonic Coda Waves in a Network of Sensors. Sensors.

[13] Hafiz A., Schumacher T. (2018). Monitoring of Stresses in Concrete Using Ultrasonic Coda Wave Comparison Technique. J. Nondestruct. Eval..

[14] Fröjd P., Ulriksen P. (2017). Frequency selection for coda wave interferometry in concrete structures. Ultrasonics.

[15] Wunderlich C., Niederleithinger E. (2011). Evaluation of Temperature Influence on Ultrasound Velocity in Concrete by Coda Wave Interferometry. Nondestructive Testing of Materials and Structures.

[16] Ju T., Li S., Achenbach J., Qu J. (2015). Effects of moisture on ultrasound propagation in cement mortar. AIP Conf. Proc..

[17] Lillamand I., Chaix J.F., Ploix M.A., Garnier V. (2010). Acoustoelastic effect in concrete material under uni-axial compressive loading. NDT & E Int..

[18] Larose E., Planes T., Rossetto V., Margerin L. (2010). Locating a small change in a multiple scattering environment. Appl. Phys. Lett..

[19] Priestley M.B. (1981). Spectral Analysis and Time Series.

[20] Roberts P.M., Phillips W.S., Fehler M.C. (1992). Development of the active doublet method for measuring small velocity and attenuation changes in solids. J. Acoust. Soc. Am..

[21] Sens-Schönfelder C., Wegler U. (2006). Passive image interferometry and seasonal variations of seismic velocities at Merapi Volcano, Indonesia. Geophys. Res. Lett..

[22] Ryzhik L., Papanicolaou G., Keller J.B. (1996). Transport equations for elastic and other waves in random media. Wave Motion.

[23] Pacheco C., Snieder R. (2005). Time-lapse travel time change of multiply scattered acoustic waves. J. Acoust. Soc. Am..

[24] Planès T., Larose E., Rossetto V., Margerin L. (2015). Imaging multiple local changes in heterogeneous media with diffuse waves. J. Acoust. Soc. Am..

[25] Branch M.A., Coleman T.F., Li Y. (1999). A Subspace, Interior, and Conjugate Gradient Method for Large-Scale Bound-Constrained Minimization Problems. SIAM J. Sci. Comput..

[26] Konertz D., Löschmann J., Clauß F., Mark P. (2019). Fiber optic sensors for continuous strain measurement in concrete. Bauingenieur.

[27] Clauß F., Epple N., Ahrens M.A., Niederleithinger E., Mark P. (2020). Comparison of Experimentally Determined Two-Dimensional Strain Fields and Mapped Ultrasonic Data Processed by Coda Wave Interferometry. Sensors.

[28] Clauß F., Ahrens M.A., Mark P. (2021). A Comparative Evaluation of Strain Measurement Techniques in Reinforced Concrete Structures—A Discussion of Assembly, Application, and Accuracy. Struct. Concr..

[29] Wang X., Chakraborty J., Niederleithinger E. (2021). Noise Reduction for Improvement of Ultrasonic Monitoring Using Coda Wave Interferometry on a Real Bridge. J. Nondestruct. Eval..

[30] Fröjd P., Ulriksen P. (2016). Continuous wave measurements in a network of transducers for structural health monitoring of a large concrete floor slab. Struct. Health Monit..

[31] Fröjd P., Ulriksen P. (2017). Detecting damage events in concrete using diffuse ultrasound structural health monitoring during strong environmental variations. Struct. Health Monit..

[32] Xue Q., Larose E., Moreau L., Thery R., Abraham O., Henault J.M. (2021). Ultrasonic monitoring of stress and cracks of the 1/3 scale mock-up of nuclear reactor concrete containment structure. arXiv.

